# SiO_2_-Induced Performance Deterioration in Magnesium Phosphate Cement: Chemical Consumption and Physical Deactivation of Reactive Magnesia

**DOI:** 10.3390/ma19071334

**Published:** 2026-03-27

**Authors:** Yuanquan Yang, Xiaoyu Ying, Jiamin Han, Chengwen Luan

**Affiliations:** 1 School of Architecture and Civil Engineering, Liuzhou Institute of Technology, Liuzhou 545616, China; 2 School of Materials Science and Engineering, Shenyang Ligong University, Shenyang 110159, China; 3 School of Materials Science and Engineering, Shenyang Jianzhu University, Shenyang 110168, China

**Keywords:** magnesium phosphate cement, SiO_2_, reactive magnesia, forsterite, calcination temperature, dual damage mechanism

## Abstract

This study investigates the dual mechanisms by which SiO_2_ deteriorates magnesium phosphate cement (MPC) performance. MgO-SiO_2_ clinkers were prepared using lightly calcined magnesia (MgO) with SiO_2_ additions ranging from 1% to 9%, followed by calcination at temperatures between 1100 °C and 1500 °C. Through XRD–Rietveld refinement, workability, compressive strength, and hydration heat analyses, the damaging effects of SiO_2_ were systematically evaluated. Results reveal that SiO_2_ degrades MPC through two concurrent mechanisms: chemical consumption and physical deactivation of reactive MgO. Chemically, SiO_2_ reacts with MgO during calcination to form inert forsterite (Mg_2_SiO_4_), irreversibly reducing reactive MgO content. Physically, SiO_2_ and its reaction products lower the crystallinity and reactivity of remaining MgO while diluting reactive components. A calcination temperature of 1200 °C was optimal, yielding the highest compressive strength (3 d strength > 30 MPa). Increasing SiO_2_ dosage monotonically reduced strength; at 1200 °C, 9% SiO_2_ reduced 3 d strength by ~40% compared to 1%. Hydration heat analysis showed that both heat flow rate and cumulative heat release increased with SiO_2_ content due to enhanced heterogeneous nucleation from Mg_2_SiO_4_. Critically, this increased heat output did not translate into higher strength, indicating that microstructural quality—not reaction extent—governs mechanical performance. Rietveld quantification confirmed that Mg_2_SiO_4_ formation increased linearly with SiO_2_ dosage and temperature (reaching 72.24% at 1500 °C with 9% SiO_2_), providing the material basis for dual damage. This work offers mechanistic insights and experimental support for utilizing low-grade magnesite and optimizing MPC performance.

## 1. Introduction

Magnesium phosphate cement (MPC), as a special cementitious material characterized by early strength, rapid hardening, high bonding strength, and excellent biocompatibility, demonstrates irreplaceable application value in fields such as rapid repair, hazardous waste solidification, and biomedicine [1,2,3]. The core driving force of its hydration reaction stems from the acid–base neutralization reaction between highly reactive magnesium oxide and acid phosphates, generating a dense gel matrix primarily composed of struvite (MgKPO_4_·6H_2_O) [4,5,6]. However, the large-scale application of MPC is constrained by its heavy reliance on high-purity, highly reactive magnesia raw materials, which are typically derived from the refinement of high-quality magnesite [7,8]. With the increasing scarcity of global high-quality magnesite resources and the rising environmental costs of mining, efficiently utilizing low-grade magnesite, which is abundant but contains higher impurity levels, has become a key bottleneck in promoting the sustainable development of the MPC industry [9,10].

Low-grade magnesite typically contains 2–15% SiO_2_ impurities [11]. While SiO_2_ has traditionally been regarded as a harmful component in MPC, its specific mechanisms of action remain poorly understood, lacking systematic and quantitative scientific explanation. This knowledge gap hinders the rational utilization of low-grade resources. If SiO_2_ merely acts as an inert filler, its effects could be compensated by mix proportion adjustments. However, if it actively participates in physical and chemical interactions during MPC preparation and hydration, it could fundamentally impact material properties [11].

Most existing research has focused on high-purity systems. Studies on impurity-containing MPC, particularly the behavior of SiO_2_ during the critical process step—high-temperature calcination for preparing reactive MgO clinker—are notably lacking. The calcination regime directly dictates the crystal structure, specific surface area, and reactivity of MgO, and the presence of impurities is highly likely to alter the phase assemblage evolution path of the clinker [12,13,14]. In Portland cement systems, excess MgO as periclase can cause volume instability, highlighting the influence of impurities on clinker mineral stability [15,16]. Within the context of MPC, questions arise: Does SiO_2_ react with MgO, and to what extent? How would such a reaction alter the reactive components of the clinker? How would these effects subsequently transfer to the hydration process and mechanical properties of the final MPC? These questions form the starting point of this study.

The role of SiO_2_ in alkaline cementitious environments is complex [17,18,19]. In alkali-activated materials and MgO-SiO_2_-H_2_O systems, silicate dissolution and formation of gel phases (e.g., magnesium silicate hydrate, M-S-H) significantly influence performance [20,21]. This suggests that within the locally high-alkalinity environment during initial MPC hydration, SiO_2_ might not be entirely inert. However, unlike the aforementioned systems, MPC is characterized by rapid reaction rates, concentrated heat release, and well-defined final products. The timing and manner of impurity intervention in this dynamic process, as well as its interference mechanism with the main reaction network, remain unclear [22]. In recent years, some scholars have begun exploring the role of SiO_2_ in the MgO-SiO_2_-K_2_HPO_4_ system, confirming the existence of silicon-containing gel phases or amorphous silicon phosphate phases [23]. These studies indicate that SiO_2_ does participate in certain chemical reactions within the MPC system, but the nature of the reaction products and their quantitative impact on system performance still lack clear definition [21,22]. Crucially, if SiO_2_ reacts with MgO during the calcination stage to form inert phases like forsterite (Mg_2_SiO_4_), this would irreversibly consume reactive raw materials and potentially alter the crystalline morphology of the remaining MgO. Such modification at the “precursor” stage would have implications throughout the entire material lifecycle, yet relevant research is nearly absent [24].

Therefore, this study moves beyond the vague notion of simply classifying SiO_2_ as a harmful impurity. It aims, through model experiments, to deeply analyze its role in the critical preparation stages of MPC. This research focuses on two core questions: (1) How does SiO_2_ alter the mineral phase composition of the clinker through reaction with MgO during calcination, and to quantitatively assess its consumption of reactive components; (2) How does this modified clinker systematically influence the subsequent hydration kinetics, fresh slurry properties, and mechanical performance of the hardened MPC. By establishing a clear correlation among “calcination regime–clinker composition–hydration behavior–macroscopic properties,” this study aims to provide direct mechanistic explanations and process guidance for the rational application of low-grade magnesite, clarify the primary contradictions leading to performance degradation, and thus promote the development of the MPC industry towards more resource-efficient utilization [25]. Unlike previous studies that primarily focused on high-purity MgO systems or the role of SiO_2_ in post-hydration phases, this work provides the first systematic quantification of how SiO_2_ alters the clinker phase assemblage during calcination—revealing that its damaging effects are “pre-programmed” into the material before hydration even begins.

## 2. Raw Materials and Test Methods

### 2.1. Raw Materials

The experiment used lightly calcined magnesia (MgO, purity 98%, prepared by light calcination of magnesite (analytical grade, Sinopharm Chemical Reagent Co., Ltd., Shanghai, China), D50 = 2.1 μm, specific surface area 350 m^2^/kg) and SiO_2_ (purity ≥ 99.5%, analytical grade, Sinopharm Chemical Reagent Co., Ltd., Shanghai, China) as the main raw materials to simulate the typical impurity composition in low-grade magnesite. Potassium dihydrogen phosphate (KH_2_PO_4_, analytical grade, Sinopharm Chemical Reagent Co., Ltd., Shanghai, China) was used as the acid phosphate component, borax decahydrate (Na_2_B_4_O_7_·10H_2_O, analytical grade) as the retarder, and deionized water as the mixing water.

### 2.2. Mix Proportion Design

(1) Raw Meal Proportion for Cement Clinker

To simulate the typical SiO_2_ content range in low-grade magnesite, six groups of MgO-SiO_2_ systems were designed. The Ref group (0% SiO_2_, 100% MgO) serves as the baseline control for evaluating the effects of SiO_2_ addition. The specific proportions are shown in Table 1.

The powders of each formulation were mixed in a planetary ball mill at 300 rpm for 30 min to ensure uniformity. Subsequently, 5 wt.% polyvinyl alcohol solution was added as a binder, and the mixture was pressed into disks of Φ50 mm × 10 mm. These disks were dried in an oven at 80 °C for 24 h and set aside.

(2) MPC Paste Mix Proportion

The calcined clinkers from each composition (MS-1 to MS-5) were used as the alkaline component. MPC pastes were prepared with a clinker-to-potassium dihydrogen phosphate mass ratio of 3:1. The water-to-binder ratio (mass of water to total solid mass) was fixed at 0.18, and the borax retarder dosage was 10% of the mass of the alkaline clinker. Based on this, for every 100 g of total MPC solid mass, the composition included 75 g clinker, 25 g KH_2_PO_4_, 7.5 g borax, and 18 g of mixing water.

### 2.3. Preparation and Calcination of Clinker

The pressed raw meal disks were placed in a high-temperature furnace (KSL-1700X, Shenyang Kejing Automation Equipment Co., Ltd., Shenyang, China) for calcination. The temperature was increased to the target temperature (1100, 1200, 1300, 1400, 1500 °C) at a rate of 5 °C/min, held for 1 h, and then allowed to cool to room temperature inside the furnace. The calcined clinker was crushed using a jaw crusher and then ground in a planetary ball mill until it all passed through a 200-mesh sieve (≤74 μm). It was then sealed and stored for later use.

### 2.4. Testing and Characterization

#### 2.4.1. Mechanical and Workability Properties

Compressive Strength: According to standard JC/T2537-2019 [26] the freshly mixed paste was poured into 20 mm × 20 mm × 20 mm steel molds and compacted by vibration. Specimens were cured at 20 ± 2 °C and relative humidity > 90% until the specified ages (4 h, 1 d, 3 d). Compressive strength was tested using a YAW-3000D testing machine (Jinan Tianchen Testing Machine Manufacturing Co., Ltd., Jinan, China) at a loading rate of 0.5 MPa/s. The result for each group was taken as the average of three specimens.

Fluidity and Setting Time: According to JC/T2537-2019, the fluidity of the fresh paste was tested using the truncated cone mold method. Setting time was determined using a Vicat apparatus, with the initial setting time defined as the time when the needle penetrated to a distance of 4 ± 1 mm from the bottom plate.

#### 2.4.2. Phase Analysis

X-ray Diffraction (XRD): XRD analysis was performed using a PANalytical X’Pert Powder diffractometer (Cu Kα radiation, λ = 1.5406 Å, Malvern Panalytical B.V., Almelo, The Netherlands) over a scan range of 10–70° (2θ) with a step size of 0.02°. Quantitative phase analysis was conducted using GSAS-II software obtained from the official GitHub repository (https://github.com/AdvancedPhotonSource/GSAS-II-buildtools/releases, accessed on 27 February 2026) for Rietveld refinement. Instrument parameters were used as a baseline, and periclase (MgO, ICSD No. 9863), forsterite (Mg_2_SiO_4_, ICSD No. 201281), and cristobalite (SiO_2_, ICSD No. 35034) were selected as initial structure models. Refined parameters included: background function (Chebyshev polynomial, 6 terms), unit cell parameters, zero shift, profile parameters (Thompson-Cox-Hastings pseudo-Voigt function), preferred orientation (March-Dollase model), and temperature factors.

The reliability of Rietveld quantitative analysis was assessed using the weighted profile R-factor (Rwp) and goodness-of-fit (GOF). Rwp values below 20% and GOF values below 2.5 are generally considered acceptable for phase quantification in cementitious materials. Potential uncertainties may arise from peak shape modeling, preferred orientation effects (corrected using the March-Dollase model), and the presence of minor amorphous phases not accounted for in the refinement. To minimize these uncertainties, consistent sample preparation and measurement protocols were followed, and three independent measurements were performed for each sample. The consistent Rwp (12.75–14.35%) and GOF (1.8–2.3) values across all samples confirm the stability and reliability of the quantitative results for comparative analysis.

#### 2.4.3. Hydration Heat Analysis

A TAM Air eight-channel isothermal calorimeter was used to continuously monitor the hydration heat evolution process of the MPC pastes over 72 h at a constant temperature of 25.0 ± 0.1 °C.

## 3. Results and Discussion

### 3.1. Regulation of Clinker Phase Composition by Calcination Temperature and SiO_2_ Content

The SiO_2_ dosage range of 1% to 9% was selected to encompass the typical impurity levels found in low-grade magnesite resources, which are reported to range from ~1% to over 17% SiO_2_ depending on the ore source [7,9]. This range allows systematic investigation of the transition from minor impurity effects to severe performance deterioration, covering the majority of SiO_2_ concentrations relevant to practical applications.

XRD patterns combined with Rietveld quantitative analysis clearly revealed the dual regulatory effects of calcination temperature and SiO_2_ content on the mineral composition of MgO-SiO_2_ system clinkers. The main crystalline phases in the clinker were periclase (MgO) and forsterite (Mg_2_SiO_4_). Unreacted cristobalite (SiO_2_) phase could be detected at high SiO_2_ dosages, with no other impurity phases formed. The quality of Rietveld refinement was good (Rwp = 12.75–14.35%, all ≤20%), confirming the reliability of the quantitative results.

**Figure 1:**
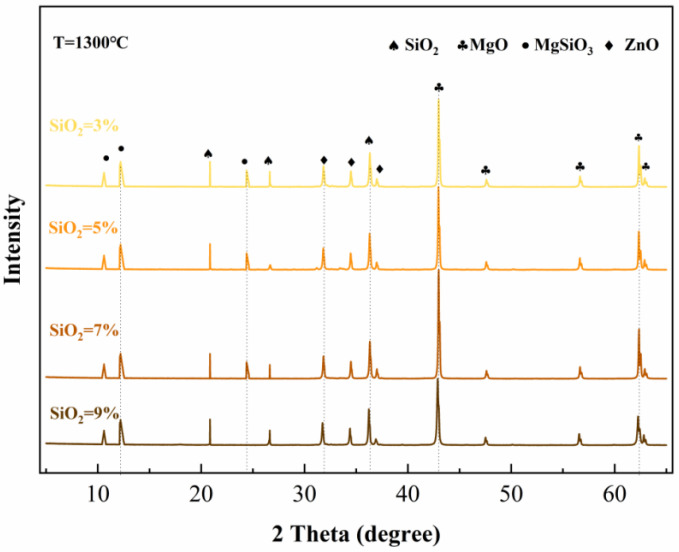
Effect of SiO_2_ content on clinker composition at 1300 °C.

SiO_2_ content was the core factor determining the phase equilibrium of the clinker. Figure 1 shows the XRD patterns and Rietveld quantitative results of clinkers with different SiO_2_ dosages calcined at 1300 °C. As the SiO_2_ content increased from 1% to 9%, the intensity of MgO diffraction peaks showed a trend of “initially increasing then decreasing”. When the SiO_2_ dosage was between 1% and 5%, the MgO diffraction peaks gradually intensified, reaching peak crystallinity at 5% dosage. When the SiO_2_ dosage exceeded 5%, the intensity of MgO diffraction peaks began to decline. This decline in diffraction intensity, coupled with the concurrent increase in Mg_2_SiO_4_ formation, provides direct crystallographic evidence for the physical deactivation mechanism: the envelopment of MgO particles by Mg_2_SiO_4_ not only consumes reactive MgO but also physically impedes MgO crystal growth and reduces the accessibility of remaining MgO surfaces to subsequent hydration. The broadening of MgO diffraction peaks (increased FWHM) at higher SiO_2_ dosages further indicates increased microstrain and reduced crystallite size—both manifestations of physical disruption to the MgO crystal structure. However, when the SiO_2_ dosage exceeded 5%, the substantial formation of Mg_2_SiO_4_ consumed MgO, and the product enveloped the surface of MgO particles, inhibiting their crystal growth, resulting in a decrease in diffraction peak intensity.

**Figure 2:**
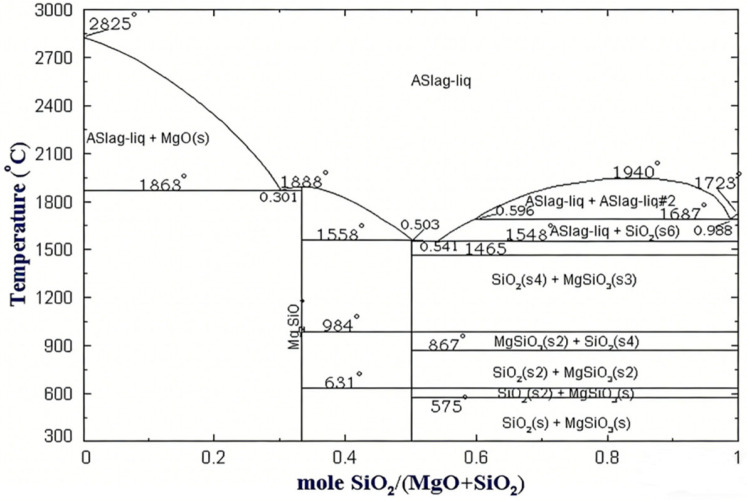
SiO_2_-MgO binary thermodynamic phase diagram [27].

Concurrently, the amount of Mg_2_SiO_4_ formed increased approximately linearly with increasing SiO_2_ content, rising from trace levels (approx. 2.3%) at 1% dosage to a significant level (approx. 35.8%) at 9% dosage. Referring to the thermodynamic phase diagram of the MgO-SiO_2_ system (Figure 2), within the experimental proportion range of this study (MgO/SiO_2_ molar ratio > 2:1), the system consistently lies within the two-phase region of MgO and Mg_2_SiO_4_. Any additional SiO_2_ will continuously react with MgO via solid-state reaction to form Mg_2_SiO_4_ until SiO_2_ becomes excessive or the reaction reaches equilibrium.

This solid-state reaction has dual negative effects. On the one hand, the “chemical consumption” effect irreversibly transforms reactive MgO into non-cementitious Mg_2_SiO_4_, reducing the absolute content of reactive components in the clinker. On the other hand, the “physical deactivation” effect occurs as the formed Mg_2_SiO_4_ coats the surface of the remaining MgO particles, potentially hindering their subsequent hydration reaction. These two effects together lay the precursor foundation for the deterioration of MPC hydration performance.

**Figure 3:**
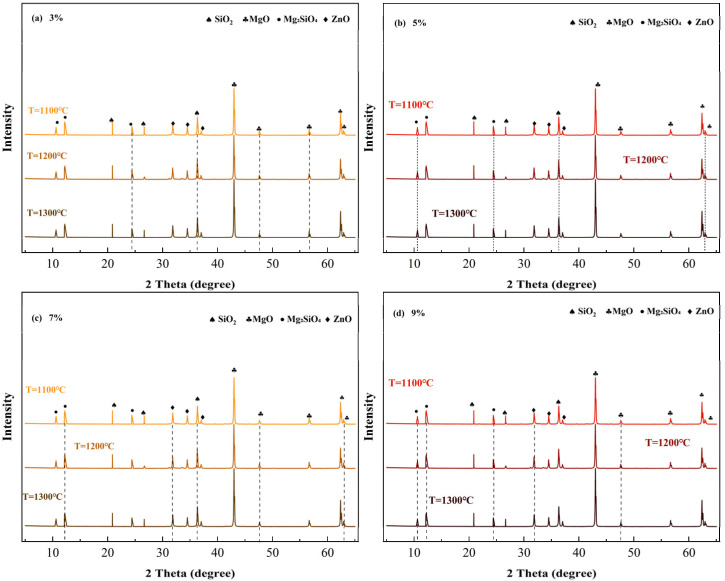
Effect of calcination temperature on clinker composition.

Calcination temperature further regulates the clinker phase composition by influencing the extent of the solid-state reaction and crystal development processes. Figure 3 displays the XRD patterns of clinkers with a fixed SiO_2_ dosage (5%) calcined at different temperatures (1100–1500 °C). As the calcination temperature increased, the intensity of Mg_2_SiO_4_ diffraction peaks continuously strengthened, and the peak shapes became sharper, indicating a deeper solid-state reaction and more perfected forsterite crystal development.

**Figure 4:**
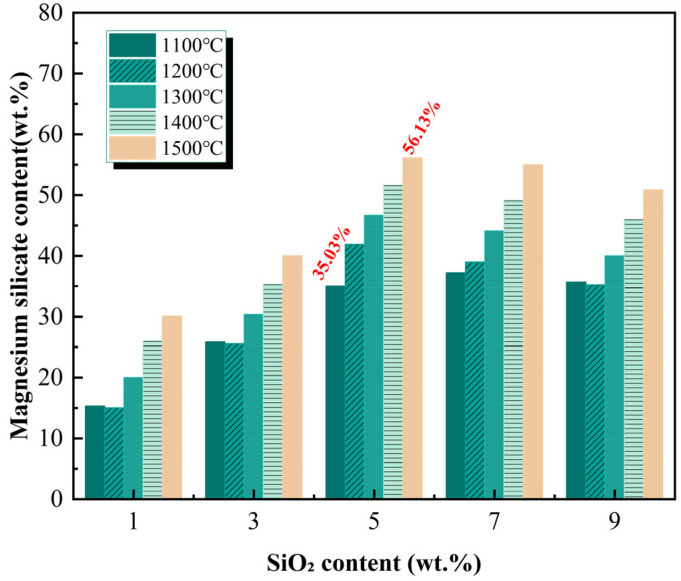
Effect of calcination temperature on forsterite content in clinker.

The Rietveld quantitative analysis results (Figure 4) further reveal that the amount of Mg_2_SiO_4_ formed increases monotonically with rising calcination temperature: approximately 18.5% at 1100 °C, increasing to 21.3% at 1200 °C, 24.8% at 1300 °C, 28.6% at 1400 °C, and reaching 32.4% at 1500 °C. This trend indicates that high temperatures provide sufficient thermodynamic driving force and kinetic conditions for the solid-state reaction between MgO and SiO_2_, promoting the reaction towards completion.

Simultaneously, the effect of high temperature on the MgO crystal structure exhibits a “double-edged sword” characteristic. On the one hand, high temperature promotes the growth of MgO grains and enhances crystallinity, resulting in a more perfect crystal structure, which benefits the physical strength of the clinker particles themselves. On the other hand, excessive sintering significantly reduces the number of reactive sites on the MgO surface—grain growth reduces specific surface area, and increased crystallinity decreases lattice defects. Both factors collectively diminish the intrinsic hydration reactivity of MgO. This phenomenon represents the intensified manifestation of SiO_2_’s “physical deactivation” effect at high temperatures.

The regulation of clinker phase composition by calcination temperature and SiO_2_ content directly establishes the material basis for SiO_2_-induced damage to MPC performance. On the one hand, the material basis for “chemical consumption” lies in the quantity of Mg_2_SiO_4_ formed from the reaction between SiO_2_ and MgO, which dictates the extent of reactive MgO consumption—higher SiO_2_ dosage and higher calcination temperature lead to greater Mg_2_SiO_4_ formation and less residual reactive MgO. On the other hand, the material basis for “physical deactivation” lies in the crystallinity and grain size of the remaining MgO, which determines the abundance of reactive sites on its surface. Although high-temperature calcination promotes Mg_2_SiO_4_ formation, it also increases the crystallinity and grain size of the residual MgO, significantly reducing surface reactive sites. Both effects are “inscribed” into the material’s microstructure during the clinker preparation stage and will comprehensively manifest their systematic influence on the workability of fresh paste, mechanical properties of hardened bodies, and hydration kinetics during subsequent MPC hydration.

It is important to clarify that while the “chemical consumption” of reactive MgO is directly and quantitatively evidenced by the increased Mg_2_SiO_4_ content from Rietveld analysis, the proposed “physical deactivation” effect operates through a combination of mechanisms that are more challenging to quantify directly. This deactivation is inferred from indirect but compelling evidence: (i) the systematic changes in MgO diffraction peak shapes and intensities (Figure 1), suggesting altered crystallinity; (ii) the monotonic increase in early-age hydration heat flow (which will be discussed in Section 3.4), which—combined with the decoupling from strength—indicates competition between heterogeneous nucleation and reduced reactive surface availability; (iii) the disproportionately severe loss of early-age (4 h) compressive strength compared to later-age (3 d) strength (which will be discussed in Section 3.2), which points towards an immediate physical hindrance of the initial rapid hydration reactions. Therefore, in the following discussion, “physical deactivation” is presented as a mechanistic model that best explains the synergistic trends observed across our multi-faceted experimental data.

### 3.2. Influence of Clinker Characteristics on the Workability of Fresh MPC Paste

The setting time and fluidity of fresh MPC paste are key workability indicators for its engineering construction application. Experimental results demonstrate that both the calcination temperature of the clinker and the SiO_2_ dosage exert a regular regulatory effect on the workability of the paste, with the influence trends of the two factors being consistent.

**Figure 5:**
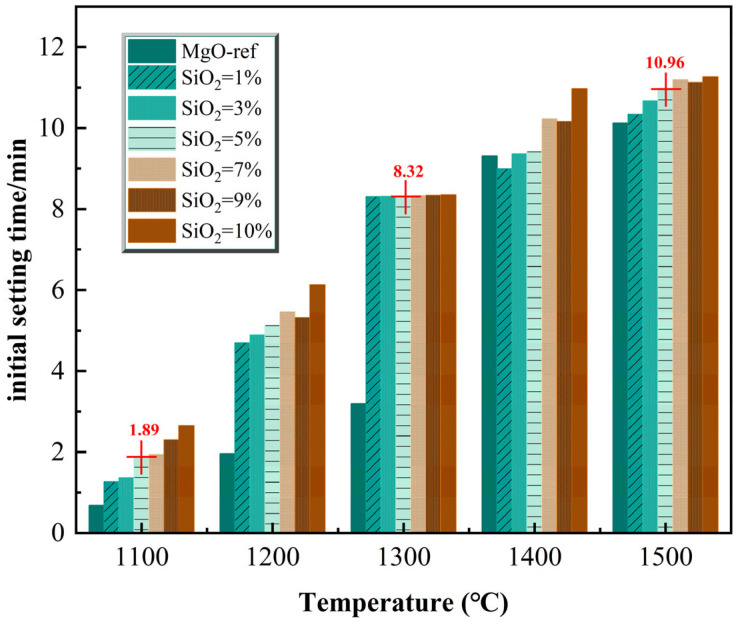
Effect of calcination temperature and SiO_2_ content on cement setting time.

Setting time test results (Figure 5) show that increasing calcination temperature and increasing SiO_2_ content both significantly delay the initial setting process of the MPC paste. Taking the clinker with 5% SiO_2_ dosage as an example, as the calcination temperature increased from 1100 °C to 1500 °C, the initial setting time extended from 1.89 min to 10.96 min; the setting time of the paste with clinker calcined at 1300 °C was approximately 6.5 min longer than that with clinker calcined at 1100 °C. The core reason for this phenomenon is the dual decrease in the content and reactivity of reactive MgO. High temperatures and high SiO_2_ dosages promote the formation of more inert Mg_2_SiO_4_ while also increasing MgO crystallinity. This leads to a significant reduction in the number of reactive sites on the clinker that can rapidly undergo acid–base neutralization with potassium dihydrogen phosphate, substantially delaying the establishment of the hydration reaction network, ultimately manifesting as prolonged setting time. Additionally, the type and concentration of phosphate can also influence setting behavior; increased potassium dihydrogen phosphate concentration may further extend the setting time of the paste, creating a synergistic effect with reduced clinker reactivity.

**Figure 6:**
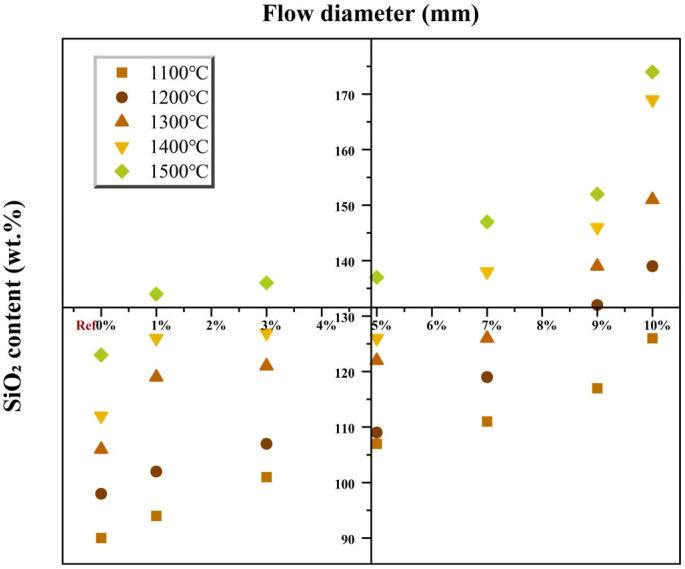
Effect of calcination temperature and SiO_2_ content on cement fluidity.

Furthermore, the SiO_2_ content significantly influenced the fluidity of MPC at different calcination temperatures. As shown in Figure 6, assuming a linear relationship between the two variables for illustration, the fluidity of the paste monotonically increased with increasing calcination temperature and SiO_2_ content, correlating well with the trend observed for setting time. This change primarily stems from two aspects: First, the reduced clinker reactivity slows down the initial hydration reaction rate upon contact with water, postponing the structuration process of the paste and diminishing flocculation among particles within the system. Second, high-temperature calcination lowers the surface energy of clinker particles, reducing their adsorption capacity for mixing water, thereby releasing more free water to lubricate the contact interfaces between particles and effectively decreasing the internal frictional resistance of the paste. It is crucial to note that this improvement in workability comes at the expense of weakened hydration reaction kinetics. The increase in free water and the decrease in reaction rate both potentially negatively affect the early strength development of MPC, manifesting as slower early strength gain.

### 3.3. Mechanical Property of Hardened MPC Paste

**Figure 7:**
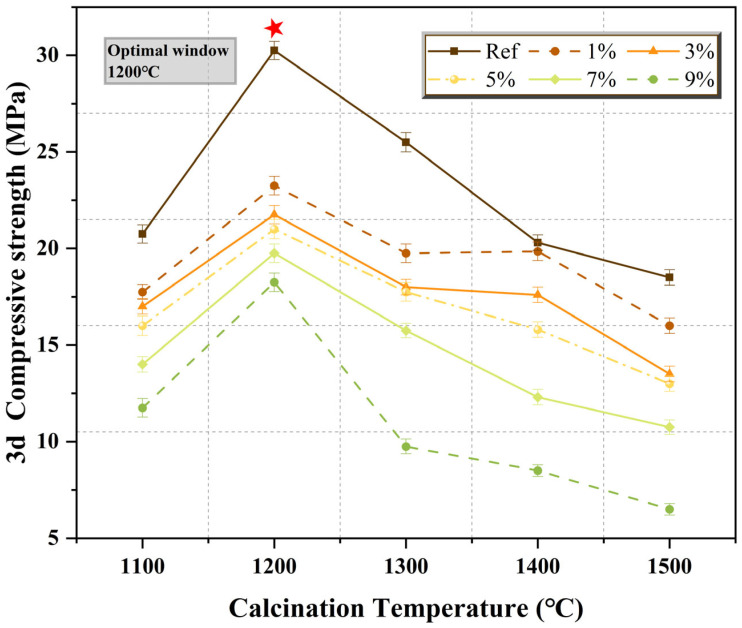
Effect of calcination temperature on 3 d compressive strength of MPC with different SiO_2_ contents.

**Figure 8:**
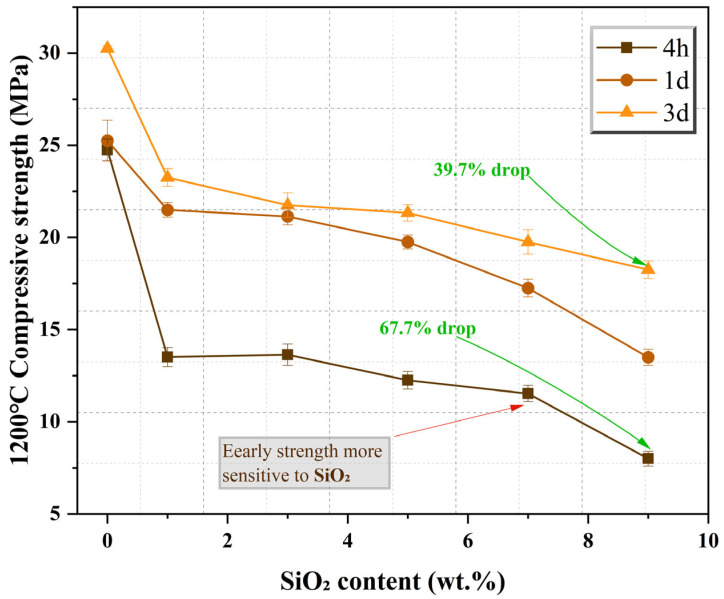
Effect of SiO_2_ content on compressive strength of MPC at different curing ages (4 h, 1 d, 3 d) under optimal calcination temperature of 1200 °C.

Compressive strength is a core performance indicator for MPC as a rapid-repair structural cementitious material. Its development pattern is subject to the coupled regulation of calcination temperature and SiO_2_ content, exhibiting differentiated responses at different ages (4 h, 1 d, 3 d). Figure 7 illustrates the distribution of 3 d compressive strength of MPC under different calcination temperatures and SiO_2_ contents, while Figure 8 presents the trend of strength at various ages as a function of SiO_2_ content at the optimal calcination temperature of 1200 °C.

Calcination temperature exhibits an optimal window effect on MPC compressive strength, and this effect is evident across all SiO_2_ content groups. As seen in Figure 7, the 3 d strength at 1200 °C calcination reached 30.25 MPa, a 45.8% increase compared to 1100 °C (20.75 MPa) and a 63.5% increase compared to 1500 °C (18.50 MPa). This pattern directly reflects the synergistic effect of the “dual damage mechanism” of SiO_2_ on reactive MgO and the calcination temperature:

① 1100 °C Low-temperature Calcination: The solid-state reaction is incomplete, resulting in insufficient Mg_2_SiO_4_ formation, and unreacted free SiO_2_ exists in the clinker. At this point, the “chemical consumption” effect of SiO_2_ on reactive MgO is weak, but the “physical deactivation” effect is already apparent—unreacted SiO_2_ physically dilutes the reactive components, and the clinker particles themselves have a low degree of sintering and poor strength. At this temperature, the 3 d strengths of all SiO_2_ content groups are moderate, ranging from 20 to 30 MPa, with a clear downward trend as SiO_2_ increases.

② 1200 °C Optimal Calcination: The solid-state reaction is sufficient and moderately progressed, with SiO_2_ and MgO reacting to form an appropriate amount of Mg_2_SiO_4_. At this temperature, the “chemical consumption” effect of SiO_2_ is controlled (sufficient reactive MgO is retained), and the “physical deactivation” effect is not yet excessive (MgO crystallinity is moderate, reactive sites are abundant). The clinker possesses both good hydration reaction kinetics and particle structure, allowing hydration products to form a dense gel network. All curves peak at this temperature, confirming 1200 °C as the optimal calcination temperature window.

③ ≥1300 °C High-temperature Calcination: High temperatures provide ample thermodynamic driving force for the solid-state reaction between MgO and SiO_2_, significantly intensifying the “dual damage mechanism” of SiO_2_. On the one hand, the “chemical consumption” effect is exacerbated—the amount of Mg_2_SiO_4_ formed becomes excessive (see Figure 4), drastically reducing the absolute content of reactive MgO. On the other hand, the “physical deactivation” effect becomes prominent—excessive sintering leads to a sharp increase in the grain size and excessive crystallinity of the remaining MgO, significantly reducing the number of reactive sites on its surface. The superposition of these dual damages results in severely insufficient hydration reaction kinetics, causing a significant decrease in strength across all SiO_2_ content groups, with the decline being more drastic for high SiO_2_ dosage groups (7%, 9%).

SiO_2_ content has a significant negative monotonic impact on MPC compressive strength, and this negative effect is particularly pronounced at early ages (4 h). As shown in Figure 8, at the optimal calcination temperature of 1200 °C, the compressive strength of MPC at all ages decreases almost linearly as the SiO_2_ content in the clinker increases from 1% to 9%: 4 h strength: decreased from 24.75 MPa in the Ref group to 8.00 MPa in the 9% dosage group, a reduction of 67.7%; 1 d strength: decreased from 25.25 MPa to 13.50 MPa, a reduction of 46.5%; 3 d strength: decreased from 30.25 MPa to 18.25 MPa, a reduction of 39.7%.

The fundamental reason for this phenomenon is the superimposed amplification of the “dual damage” effect with increasing SiO_2_ dosage. On the one hand, the “chemical consumption” effect intensifies with increasing SiO_2_—more reactive MgO is converted into non-cementitious Mg_2_SiO_4_, irreversibly reducing the total amount of reactive components participating in the hydration reaction. On the other hand, the “physical deactivation” effect synchronously intensifies—unreacted SiO_2_ and formed Mg_2_SiO_4_ physically dilute and encapsulate the remaining reactive MgO, while the synergistic effect of high temperature increases the crystallinity of the remaining MgO, reducing its surface reactivity. The dual damage ultimately leads to insufficient formation of cementitious products in the hardened paste, a loose structure, and a continuous decline in compressive strength. Notably, the strength loss at moderate SiO_2_ levels (3~5%) observed in this study is comparable to or less severe than that reported for MPC containing other impurities such as iron tailings [2] or nano-silica [22], suggesting that SiO_2_ is not uniquely detrimental when its dual effects are understood and controlled. The identification of 1200° Cas, the optimal calcination temperature, also provides a processing window that previous studies, which often focused on a single calcination temperature, did not systematically explore [7,8].

It is particularly noteworthy that early-age strength is more sensitive to SiO_2_ content. The magnitude of decrease in 4 h strength (67.7%) is significantly higher than that in 3 d strength (39.7%). This indicates that the “physical deactivation” effect of SiO_2_ is more pronounced in the very early stages of hydration—the encapsulation of reactive MgO surfaces by inert phases or increased crystallinity directly hinders the rapid progress of the acid–base neutralization reaction initially, while later slow hydration can still partially supplement the cementitious products. This phenomenon also explains why high SiO_2_ dosage groups, although capable of enhancing the early heat flow rate through heterogeneous nucleation (see Section 3.4), cannot translate this into corresponding early strength. This heightened sensitivity of very early-age strength to SiO_2_ content provides the strongest macroscopic evidence for the “physical deactivation” mechanism, as chemical consumption alone would be expected to reduce strength proportionally across all ages, rather than exhibiting this pronounced age-dependent effect. This macroscopic evidence is further corroborated by the crystallographic observations presented in Section 3.1, where systematic changes in MgO peak intensities and peak broadening directly indicated compromised surface reactivity and structural disruption of the remaining MgO—the microstructural origins of physical deactivation.

MPC compressive strength exhibits a clear age-dependent increase, with all formulation groups showing significantly higher 3 d strength compared to 1 d and 4 h strength. However, the magnitude of age-dependent increase is significantly regulated by SiO_2_ content: Low SiO_2_ dosage groups (Ref, 1%, 3%): High strength growth potential, with an absolute increase from 4 h to 3 d strength of 10–15 MPa and a relative increase of 22–36%. High SiO_2_ dosage groups (7%, 9%): Limited strength growth potential, with an absolute increase from 4 h to 3 d strength of only 6–10 MPa. Although the relative increase is high (75–128%), the absolute strength values remain far lower than those of the low-dosage groups.

Taking the 1200 °C group as an example, the Ref group had a 4 h strength of 24.75 MPa and a 3 d strength of 30.25 MPa, an increase of 22.2%. In contrast, the 9% dosage group had a 4 h strength of only 8.00 MPa and a 3 d strength of 18.25 MPa, an absolute increase of 10.25 MPa, but its 3 d strength was still less than 75% of the Ref group’s 4 h strength. This indicates that although high SiO_2_ dosage reduces the total amount of reactive MgO through “chemical consumption,” the remaining reactive MgO can still continue to hydrate. However, limited by the insufficient total amount of reactive components, the final strength remains difficult to elevate to application requirements.

This pattern further confirms that the continuous progress of the hydration reaction constantly supplements cementitious products and optimizes the microstructure of the hardened paste, while the retention of highly reactive MgO (i.e., mitigating the dual damage of SiO_2_) provides the material basis for the sustained hydration reaction. Therefore, controlling the SiO_2_ dosage (recommended not exceeding 5%) is crucial for ensuring the mechanical properties of MPC in practical applications.

### 3.4. Hydration Kinetics Process

**Figure 9:**
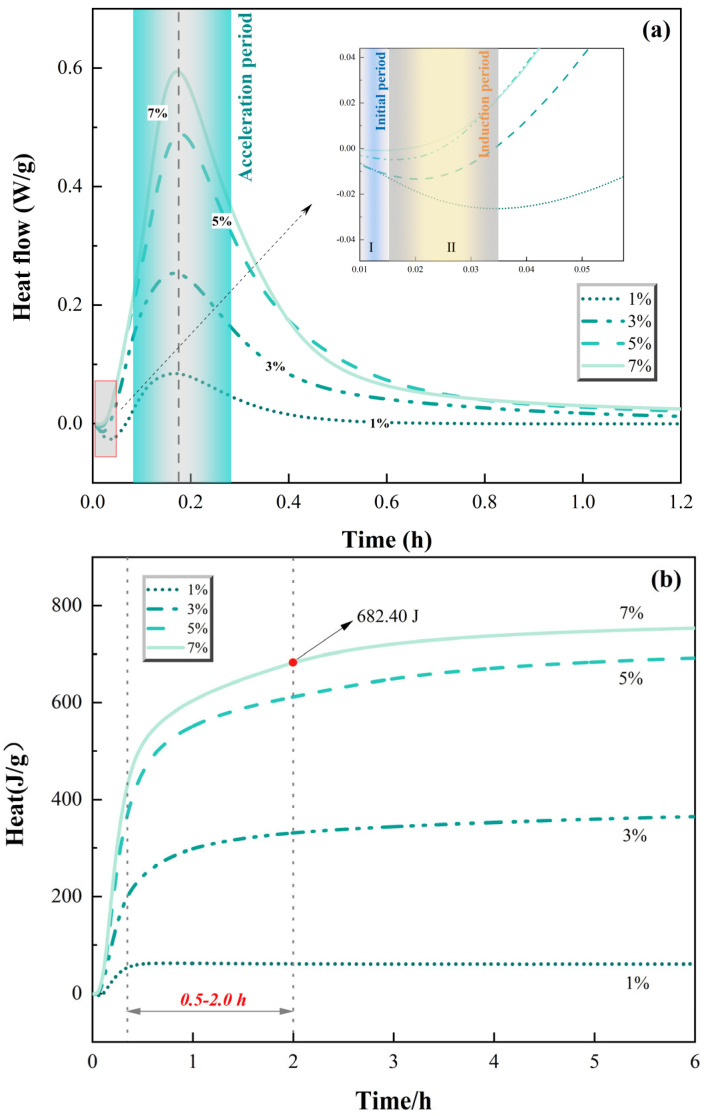
Hydration heat flow rate and cumulative heat release of MPC with different SiO_2_ contents at 1200 °C: (**a**) Hydration heat flow rate, (**b**) Cumulative heat release.

**Figure 10:**
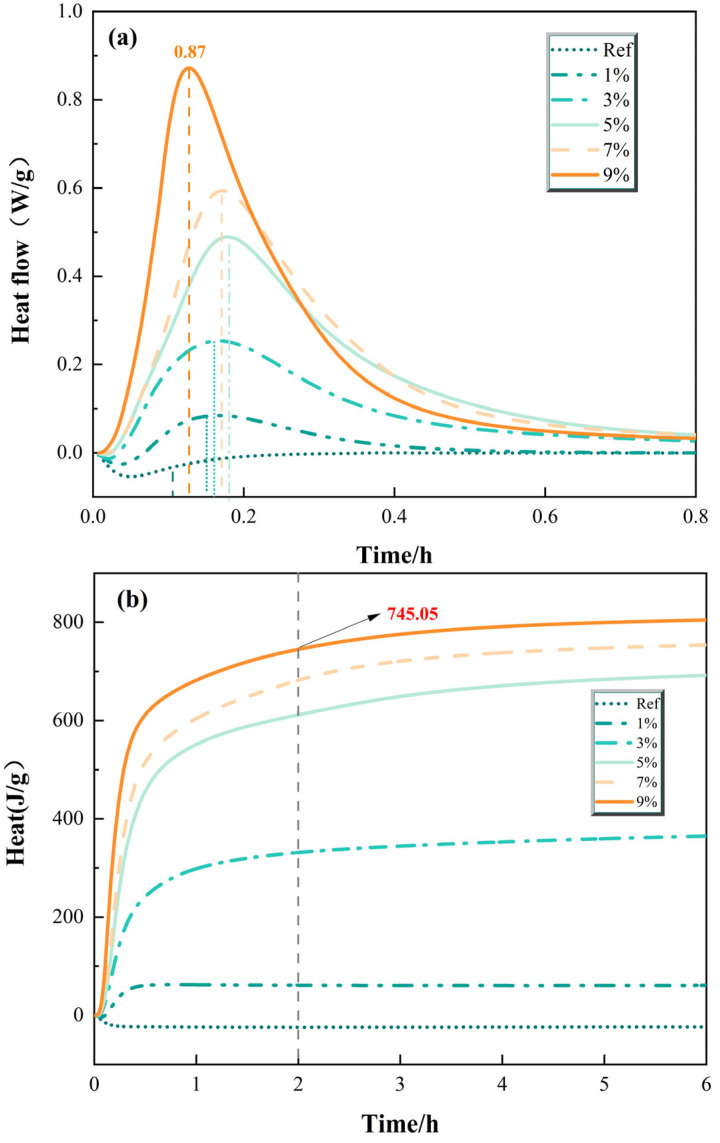
Hydration heat flow rate and cumulative heat release of MPC with different SiO_2_ contents at 1300 °C: (**a**) Hydration heat flow rate, (**b**) Cumulative heat release.

To further elucidate the influence of SiO_2_ on the reaction mechanisms of MPC, the hydration heat evolution of pastes prepared from clinkers calcined at 1200 °C and 1300 °C was monitored using isothermal calorimetry. The heat flow rate curves and cumulative heat release are presented in Figure 9 and Figure 10.

Figure 9a shows the hydration heat flow rates of MPC prepared with clinkers containing 1%, 3%, 5%, and 7% SiO_2_ calcined at 1200 °C. All pastes exhibited the typical rapid exothermic characteristic of MPC, with heat flow rising sharply upon contact with water and reaching a peak within minutes. The maximum heat flow rate increased progressively with increasing SiO_2_ content: the 1% group exhibited a peak of 0.084 W/g, while the 3%, 5%, and 7% groups reached 0.254 W/g, 0.489 W/g, and 0.594 W/g, respectively.

This progressive enhancement of the early-age heat flow rate provides direct evidence for the heterogeneous nucleation effect of SiO_2_-derived phases. As demonstrated by the Rietveld quantitative analysis in Section 3.1, higher SiO_2_ dosages lead to increased formation of forsterite (Mg_2_SiO_4_) during calcination. These Mg_2_SiO_4_ particles, along with any unreacted SiO_2_, serve as effective nucleation substrates for hydration products (primarily K-struvite), accelerating the early-stage acid–base reaction and resulting in higher peak heat flow rates.

The cumulative heat release over 72 h (Figure 9b) also increased monotonically with SiO_2_ content. The 1% group released approximately 61 J/g over 72 h, while the 3%, 5%, and 7% groups released progressively higher amounts, with the 7% group reaching nearly 682 J/g at 2 h. This trend indicates that the overall extent of hydration reaction increases with SiO_2_ dosage, despite the “chemical consumption” of reactive MgO through forsterite formation during calcination.

This apparent paradox—increased total heat release despite reduced absolute content of reactive MgO—can be reconciled by considering two factors operating in concert: First, the heterogeneous nucleation effect not only accelerates early kinetics but also promotes more complete hydration of the remaining reactive MgO. The abundant nucleation sites provided by Mg_2_SiO_4_ particles enable more efficient utilization of the available reactive surface, allowing hydration to proceed more extensively than in systems with fewer nucleation sites. Second, the physical dilution effect of inert phases (Mg_2_SiO_4_ and any unreacted SiO_2_) may increase the overall accessible surface area of the clinker. These inert particles create a more fragmented microstructure upon grinding, allowing the phosphate solution to penetrate more effectively and access reactive MgO surfaces that might otherwise remain encapsulated in a denser clinker particle.

Figure 10 presents the hydration heat characteristics of MPC prepared with clinkers containing 1% to 9% SiO_2_ calcined at 1300 °C. The same monotonic trends were observed: both the maximum heat flow rate and the cumulative heat release increased progressively with increasing SiO_2_ dosage.

The maximum heat flow rate increased from 0.08 W/g for the 1% group to 0.25 W/g, 0.49 W/g, 0.59 W/g, and 0.87 W/g for the 3%, 5%, 7%, and 9% groups, respectively. The cumulative heat release over 72 h followed a parallel trend, with the 9% group reaching approximately 745 J/g—the highest value observed across all formulations in this study.

Compared to the 1200 °C series, all groups at 1300 °C exhibited comparable or slightly lower total heat release values for equivalent SiO_2_ dosages up to 7%, consistent with the enhanced formation of inert forsterite and increased MgO crystallinity at higher temperature (Section 3.1). Nevertheless, the persistent monotonic increase with SiO_2_ content confirms that the nucleation-enhancing effect of Mg_2_SiO_4_ remains operative even when the baseline clinker reactivity is reduced by high-temperature calcination. The continued increase up to 9% SiO_2_ at 1300 °C further underscores that the relationship between SiO_2_ content and hydration extent is robust across the entire dosage range studied.

The hydration heat characteristics provide critical insights into the strength development patterns discussed in Section 3.3. The monotonic increase in both heat flow rate and cumulative heat release with SiO_2_ content observed here contrasts with the behavior reported in some alkali-activated slag systems, where SiO_2_ impurities can retard early hydration [20,21]. This difference likely reflects the distinct reaction mechanisms: in MPC, the rapid acid–base reaction is dominated by phosphate dissolution and struvite precipitation, where Mg_2_SiO_4_ acts primarily as a nucleation substrate rather than a reactive participant. Furthermore, the decoupling between heat release and strength observed at high SiO_2_ dosages is a phenomenon not previously documented in MPC literature; most prior studies have implicitly assumed that higher heat release correlates with better mechanical performance [22]. This finding challenges that assumption and highlights the importance of microstructural development beyond simple reaction extent. Perhaps the most significant finding of this study is the pronounced decoupling between total heat release and compressive strength at high SiO_2_ dosages. At 1200 °C, the 7% SiO_2_ group exhibited nearly 11 times the total heat release of the 1% group (682 J/g vs. 61 J/g over 72 h), yet its 3 d compressive strength (18.25 MPa) was only 60% of the 1% group’s strength (30.25 MPa). This striking disparity demonstrates that the sheer extent of hydration reaction—as measured by heat output—is not the primary determinant of mechanical performance in these systems.

This decoupling can be understood through the lens of the dual damage mechanism proposed in this study: At low SiO_2_ dosages (1%, 3%), the hydration reaction proceeds on highly reactive MgO surfaces with fewer but more effective nucleation sites. The limited number of Mg_2_SiO_4_ particles allows for controlled nucleation and growth, promoting the formation of a dense, interconnected gel network with superior mechanical properties. At high SiO_2_ dosages (7%, 9%), the abundant Mg_2_SiO_4_ particles trigger rapid, widespread nucleation across numerous sites. While this accelerates early kinetics and increases total heat output, the resulting hydration products precipitate in a more dispersed manner, forming a porous, less cohesive microstructure. This aligns with the “physical deactivation” mechanism: although Mg_2_SiO_4_ promotes nucleation, the coating of MgO surfaces by these inert phases and the increased crystallinity of the remaining MgO hinder the formation of a dense, load-bearing gel network. The hydration heat analysis thus reinforces the central thesis of this work: chemical consumption reduces the absolute quantity of reactive MgO, while physical deactivation compromises the microstructural quality of the hydration products formed from the remaining MgO. The combination of these dual effects explains why increased SiO_2_ content—despite enhancing early kinetics and total heat release—ultimately degrades the mechanical performance of MPC.

## 4. Conclusions

This study systematically investigated the dual mechanisms by which SiO_2_ deteriorates magnesium phosphate cement (MPC) performance. The following conclusions are drawn:

(1) Higher SiO_2_ dosage and calcination temperature promote forsterite (Mg_2_SiO_4_) formation while reducing reactive periclase. At 1500 °C with 9% SiO_2_, periclase drops to 25.76% and forsterite reaches 72.24%. High temperature also increases MgO crystallinity, reducing reactive sites and intensifying physical deactivation.

(2) Increasing temperature and SiO_2_ dosage delays setting and improves fluidity—at 5% SiO_2_, setting time at 1500 °C is ~5.8 times that at 1100 °C. This results from reduced reactive MgO content and activity, slowing hydration kinetics at the expense of early strength.

(3) Compressive strength: 1200 °C is optimal, yielding peak 3 d strength (30.25 MPa). Below 1100 °C, reaction is incomplete; above 1300 °C, excessive deactivation occurs. Strength decreases nearly linearly with increasing SiO_2_ dosage. Early-age strength is more sensitive: at 1200 °C, 4 h strength loss (67.7%) far exceeds 3 d loss (39.7%), confirming that physical deactivation dominates initial hydration.

(4) Heat flow and cumulative release increase with SiO_2_ content, evidencing heterogeneous nucleation by Mg_2_SiO_4_. Crucially, more heat does not mean higher strength—the 7% SiO_2_ group released ~11 times more heat than the 1% group but achieved only 60% of its 3 d strength. This decoupling shows that microstructural quality, not reaction extent, governs strength; abundant nucleation at high SiO_2_ produces porous gel networks.

(5) SiO_2_ damages MPC via chemical consumption (forsterite formation irreversibly reducing reactive MgO) and physical deactivation (evidenced by disproportionate early strength loss, altered kinetics, and increased MgO crystallinity). These coupled effects, originating during clinker preparation, govern MPC performance throughout its lifecycle.

This study offers actionable guidance for industrial MPC production using low-grade magnesite: (i) Optimal calcination: Maintain kiln temperature at 1200 ± 50 °C to balance MgO reactivity and forsterite formation; (ii) SiO_2_ tolerance: Raw materials with >5% SiO_2_ require beneficiation or blending to avoid >40% strength loss; (iii) Process awareness: Physical deactivation by SiO_2_ cannot be reversed by simple dilution—source control is essential. These findings provide a scientific foundation for raw material selection and process optimization in sustainable MPC manufacturing.

## Figures and Tables

**Table 1:** Raw meal proportions of MgO-SiO_2_ system (mass fraction/%).

No.	MgO	SiO_2_
Ref	100	0
MS-1	99	1
MS-2	97	3
MS-3	95	5
MS-4	93	7
MS-5	91	9

## Data Availability

The original contributions presented in this study are included in the article. Further inquiries can be directed to the corresponding author.
